# Prenatal paternal depression, anxiety, and somatic symptom burden in different risk samples: an explorative study

**DOI:** 10.1007/s00404-022-06612-2

**Published:** 2022-05-24

**Authors:** Magdalena Zacher, Nele Wollanka, Christina Sauer, Kathrin Haßtenteufel, Stephanie Wallwiener, Markus Wallwiener, Imad Maatouk

**Affiliations:** 1 Department of General Internal Medicine and Psychosomatics, University of Heidelberg, Heidelberg, Germany; 2 Department of Obstetrics and Gynecology, University of Heidelberg, Heidelberg, Germany; 3 Section of Psychosomatic Medicine, Psychotherapy and Psychooncology, Department of Internal Medicine II, Julius-Maximilian University Würzburg, Würzburg, Germany

**Keywords:** Prenatal paternal depression, Anxiety, Somatic symptom burden, Risk pregnancy, Hospitalization

## Abstract

**Purpose:**

Growing evidence implies that transition to parenthood triggers symptoms of mental burden not only in women but likewise in men, especially in high-risk pregnancies. This is the first study that examined and compared the prevalence rates of depression, anxiety, and somatic symptom burden of expectant fathers who face different risk situations during pregnancy.

**Methods:**

Prevalence rates of paternal depression (Edinburgh postnatal depression scale), anxiety (generalized anxiety disorder seven), and somatic symptom burden (somatic symptom scale eight) were examined in two risk samples and one control group in the third trimester of their partners’ pregnancy: risk sample I (*n* = 41) consist of expectant fathers whose partners were prenatally hospitalized due to medical complications; risk sample II (*n* = 52) are fathers whose partners were prenatally mentally distressed; and control group (*n* = 70) are those non-risk pregnancies.

**Results:**

On a purely descriptive level, the data display a trend of higher symptom burden of depression, anxiety, and somatic symptoms in the two risk samples, indicating that expectant fathers, whose pregnant partners were hospitalized or suffered prenatal depression, were more prenatally distressed. Exploratory testing of group differences revealed an almost three times higher prevalence rate of anxiety in fathers whose partner was hospitalized (12.2%) compared to those non-risks (4.3%).

**Conclusion:**

Results underline the need for screening implementations for paternal prenatal psychological distress, as well as specific prevention and treatment programs, especially for fathers in risk situations, such as their pregnant partners’ prenatal hospitalization.

The study was registered with the German clinical trials register (DRKS00020131) on 2019/12/09.

## Introduction

### Perinatal period as a risk factor for psychological and somatic strain in expectant fathers

The transition to fatherhood is not only an auspicious but also mentally and physically challenging process in some men [1, 2]. Compared to the depression (4.4%) and anxiety disorders (3.6%) prevalence rates worldwide [3], these prevalence rates are notably higher during the transition to parenthood. Pre and postnatal depression approximately affects 8.4% of men [4]; the prevalence of any prenatal anxiety disorder ranges from 4.1 to 16% and 2.4 to 18% in the postnatal period, depending on study methodologies and sub-syndromic symptom burden assessment [5, 6]. In addition, a range of studies indicates that expectant fathers react with higher levels of somatic symptom burden to their partners’ pregnancy as a manifestation of psychological distress on a somatic level [1, 7, 8], indicating that the transition to parenthood is a strong risk factor for mental and somatic strain.

### Negative consequences of paternal perinatal distress

The number of negative consequences of untreated paternal perinatal distress on the whole family system underlines the need for intensive research and healthcare system considerations [9]. Untreated paternal prenatal depression and anxiety are the strongest predictors for paternal postnatal depression [10], associated with maternal perinatal depression [11] and leads to a range of adverse outcomes on the psycho-social development and mental health of children [10, 12, 13].

Given these inevitable negative effects of paternal perinatal distress, studies are to detect risk samples of distressed expectant fathers to provide fast and proper psycho-social support.

### Women’s inpatient treatment due to pregnancy-related complications as possible risk factor for perinatal paternal mental health

Peripartal paternal distress studies sharply increased during the last decades; however, a limited number of studies examined the relationship between high-risk pregnancies and paternal prenatal mental health [14, 15, 46]. Partners of women hospitalized due to medically complicated pregnancies are facing several additional distressing factors compared to non-risk pregnancy. They do not only maintain their jobs and provide emotional support to their pregnant partner but also manage household demands and take care of other children. These demands are accompanied by fear and worries about the emotional and physical health of their partner and their baby [14, 16, 17]. Consequently, qualitative studies show that expectant fathers with partners in high-risk pregnancies experience crises-like emotions ranging from shock and anxiety to feelings of isolation and overwhelmed with responsibilities [16–20]. Expectant fathers feel depressive symptoms, such as exhaustion, inability to recover or sleep in the evening, and concentration reduction, due to high-stress levels [16]. Only one quantitative study investigated prenatal paternal distress in medically high-risk pregnancy samples, indicating that fathers whose partners were hospitalized due to preeclampsia or preterm premature rupture of membranes (*n* = 51) had the same risk for depression and posttraumatic stress disorder compared to expectant fathers of non-risk pregnancy (*n* = 34) [15].

The present study aimed to fill the research gap by providing more insights into prenatal paternal mental distress in different pregnancy-related risk situations.

### Aim of the study

To the best of our knowledge, this is the first study to compare the effects of different risk pregnancies on prenatal paternal mental health. This explorative study aimed to examine and compare the prevalence rates of depression, anxiety, and somatic symptom burden of expectant fathers of two potentially high-risk samples: first, expectant fathers whose pregnant partners had inpatient treatment due to pregnancy-related complications and second, risk sample that comprises expectant fathers whose pregnant partners suffered from prenatal depression. Several studies revealed that prenatal maternal depression is associated with higher rates of paternal pre- and postnatal depression, thus indicated as a risk factor for paternal perinatal mental distress [11, 21, 22, 45].

This study will provide clinical implications for a specified screening and treatment for expectant fathers by comparing different risk samples on the psychological and somatic strain.

## Materials and methods

### Design

A cross-sectional explorative study design was employed to investigate the prevalence of prenatal paternal depression, anxiety and somatic symptom burden in two different risk samples and a non-risk control group.

### Ethics

Ethical approval was granted in October 2019 from the ethics committee of the medical faculty in Heidelberg (S-641/2019). The study was registered with the German clinical trials register (DRKS00020131).

### Procedure and participants

**Fig. 1:**
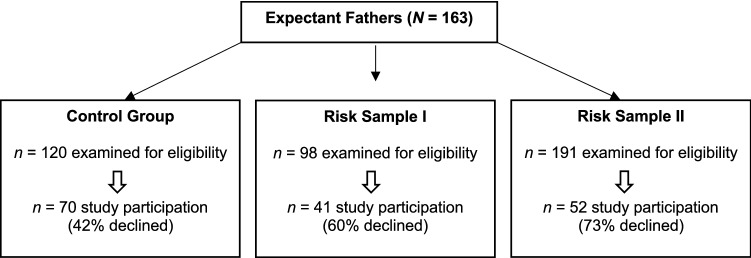
Sample description

The study was conducted from October 2019 to August 2020 in the department of obstetrics and gynecology of the university hospital of Heidelberg. The prenatal sample of expectant fathers consisted of two risk samples and a non-risk control group. A total of 163 expectant fathers were recruited in the third trimester (> 28th week) of their partners’ pregnancy. Expectant fathers and their pregnant partners were both, personally or via mail, informed about the study. Pregnant partners were asked to consent to the men’s study participation before they were asked for consent and given the set of questionnaires. General exclusion criteria are age below 18 years and lacking command of the German language (Fig. 1).

The control group (no risk pregnancy) included 70 expectant fathers whose partner and/or fetus was not suffering from serious medical conditions, without inpatient treatment due to medical complications during pregnancy. These expectant fathers were recruited in the department of obstetrics and gynecology of the university hospital of Heidelberg while attending prenatal care together with their pregnant partners or via the information evenings for giving birth in the clinic.

The risk sample I (high medical risk pregnancy) included 41 expectant fathers whose pregnant partners had inpatient treatment in the department of obstetrics and gynecology of the university hospital of Heidelberg due to pregnancy-related medical complications (e.g., cervical insufficiency). These expectant fathers were recruited while visiting their pregnant partners in the hospital.

The risk sample II (perinatal distressed pregnant partners) included 52 expectant fathers whose pregnant partner was psychologically distressed. Inclusion criteria are the men’s partner’s prenatal depression score (> 9) on the Edinburgh postnatal depression scale (EPDS) [23]. Exclusion criteria are expectant fathers whose pregnant partner and/or fetus were suffering from serious medical conditions and/or had inpatient treatment due to pregnancy-related medical complications. Expectant fathers were recruited via their pregnant partners who took part in an out-patient screening and health care program for women suffering prenatal depression in the department of obstetrics and gynecology of the university hospital of Heidelberg (mind: pregnancy program) [24].

### Measures

Following a demographic and pregnancy-related information sheet, the men received the questionnaire set containing the German versions of symptom measure scales: EPDS, generalized anxiety disorder seven (GAD-7), and somatic symptom scale eight (SSS-8). Socio-demographic data of participants included items on ethnicity, educational level, employment, relationship status, and the number of previous children. Information about the partners’ pregnancy included weeks of gestation, prenatal complications or risk factors (e.g., cervical insufficiency and pathological cardiotocography), need for inpatient treatment, multiple pregnancies, and previous pregnancy loss. Further, actual or former mental illness and actual physical illness were assessed using the following items: “Do you currently suffer from mental (physical) illness?”, “Did you ever suffer from mental illness?” and “Are you currently receiving psychotherapeutic/psychiatric treatment?”

### EPDS

The EPDS [23] was used to measure paternal depression, which is a ten-item self-rating scale with four responses scored from 0 to 3 with a maximum score of 30. The scale was originally developed to detect depression in women in the postnatal period but is also validated and often used in the screening of depression in men in the perinatal period [25, 26]. A German translation of the EPDS was validated on women in the postpartal period yielding good psychometric results [42]. Matthey et al. [25] showed that a cutoff score of ten and above was optimal to detect minor and major depression in fathers with a 71.4% sensitivity and a 93.8% specificity. Internal consistency (Cronbach’s standardized alpha) of the EPDS was 0.80 in this sample, which is comparable to that obtained by Matthey et al. [25] (*α* = 0.81).

### GAD-7

The GAD-7 [27] was used to assess prenatal paternal anxiety. It is a well-validated self-rating scale for GAD with four response options ranging from 0 to 3 with a maximum score of 21. Spitzer et al. [27] postulated cutoff points of 5, 10, and 15 for mild, moderate, and severe levels of anxiety symptoms, respectively. With a cutoff score of ten or above, the GAD-7 yielded an 89% sensitivity and 82% specificity for GAD [27]. The GAD-7 was validated for male and female German general population revealing good internal consistency (*α* = 0.89) [43] as well as for the perinatal period in women [28]. The reliability analysis showed a good internal consistency of the GAD-7 in this sample (*α* = 0.83).

### SSS-8

The SSS-8 [29] was used to measure somatic symptom burden. On eight items with five response options from 0 to 4 with a maximum score of 32, respondents evaluated how much they were bothered in the last 7 days on the following somatic symptoms: (1) stomach or bowel problems, (2) back pain, (3) pain in your arms, legs, or joints, (4) headaches, (5) chest pain or shortness of breath or dizziness, (6) feeling tired or having low energy, and (7) trouble sleeping. Gierk et al. [29] classified five groups of somatic symptom burden severity: no to minimal (0–3), low (4–7), medium (8–11), high (12–15), and very high (16–32). The scale is validated for male and female German general population [29]. Internal consistency of the SSS-8 was acceptable in this sample (*α* = 0.75) but was lower than that obtained by Gierk et al. [29] (*α* = 0.81).

### Statistical analysis

The Statistical Package for Social Science (IBM SPSS v. 25) was used for all study analyses. Non-parametric tests for group differences (Kruskal–Wallis test) were used as normal distribution assumption was violated and the aim was to only test group differences on an exploratory and purely descriptive approach. Means and medians were used for descriptive statistics due to the left-skewed distribution of the dependent variables.

## Results

Socio-demographic characteristics and parameters about the pregnancy and the men’s health are presented in Table 1.

**Table 1:** Socio-demographic properties of participants and parameters about pregnancy

Variables	All	Control group	Risk sample I	Risk sample II
Average age, *M *(SD)	34.77 (5.93)	34.29 (5.51)	34.56 (6.91)	35.58 (5.68)
Week of gestation, *M* (SD)	32.96 (3.62)	33.82 (3.57)	33.56 (3.43)	31.30 (3.39)
Education				
Elementary	7.3% (10)	9.4% (5)	7.9% (3)	4% (2)
Middle	23.7% (36)	26.6% (17)	26.3% (10)	18% (9)
High	66.4% (101)	62.5% (40)	60.6% (23)	76% (38)
Employed	97.5% (159)	97.1% (68)	100% (41)	96.1% (50)
Men’s health				
Current physical disease	6.7% (11)	2.9% (2)	9.8% (4)	9.6% (5)
Current mental illness	1.2% (2)	0% (0)	0% (0)	3.8% (2)
Former mental illness	5.5% (9)	4.3% (3)	4.9% (2)	7.7% (4)
Number of children				
First-time fatherhood	55.2% (90)	51.4% (36)	61% (25)	55.8% (29)
1 child	36.2% (59)	35.7% (25)	31.7% (13)	40.4% (21)
> 1 child	8.6% (14)	12.9% (9)	7.3% (3)	3.8% (2)
Pregnancy				
Week of gestation, *M *(SD)	32.96 (3.62)	33.82 (3.57)	33.56 (3.43)	31.30 (3.39)
Twin pregnancy	8% (13)	11.4%	9.8%	1.9%
Former abortion or stillbirth	27% (44)	25.7% (18)	31.7% (13)	25% (13)

Frequencies in percent and the total number of participants

### Prevalence and exploratory testing of group differences

Prevalence rates for depression, anxiety, and somatic symptom burden are presented in Table 2. Table 3 shows the test statistics of exploratory testing of group differences; *p* values are only interpreted on a purely descriptive approach.

**Table 2:** Prevalence of depression, anxiety, and somatic symptom burden

	All (*N* = 163) (%)	Control group (*n* = 70) (%)	Risk sample I (*n* = 41) (%)	Risk sample II (*n* = 52) (%)
EPDS (cutoff ≥ 10)	14.1 (23)	11.4 (8)	19.5 (8)	13.5 (7)
GAD-7				
Moderate (cutoff ≥ 10)	6.7 (11)	4.3 (3)	12.2 (5)	5.8 (3)
SSS-8				
Medium (cutoff ≥ 8)	16.6 (27)	10 (7)	19.5 (8)	23.1 (12)
High (cutoff ≥ 12)	11 (18)	10 (7)	7.3 (3)	15.4 (8)

Frequencies in percent and the total number of participants

**Table 3:** Descriptive and test statistics (Kruskal–Wallis) of symptom measures EPDS, GAD-7, and SSS-8

	All (*N* = 163)	Sample 1 (*n* = 70)	Sample 2 (*n* = 41)	Sample 3 (*n* = 52)	*H *(*χ*^2^)	*p*
EPDS Mdn (min–max) *M* (SD)	4 (0*–*22) 4.94 (4.21)	3 (0*–*14) 4.17 (3.71)	4 (0*–*22) 6.20 (5.32)	4 (0*–*13) 5.00 (3.65)	4.21	0.122
GAD-7 Mdn (min–max) *M* (SD)	3 (0*–*14) 3.58 (3.32)	2 (0*–*13) 2.87 (3.03)	4 (0*–*14) 4.63 (3.79)	3 (0*–*12) 3.69 (3.13)	7.42	0.025
SSS-8 Mdn (min–max) *M* (SD)	5(0*–*21) 5.53 (4.38)	5 (0*–*19) 5.30 (4.39)	4 (0*–*21) 5.20 (4.41)	5 (0*–*16) 6.10 (4.38)	1.58	0.453

#### Depression

Paternal depression was most prevalent in expectant fathers whose partners had inpatient treatment due to pregnancy-related complications and followed by fathers-to-be whose partners were prenatally distressed. The lowest depression rate was measured for fathers whose partners had non-risk pregnancies (see Table 2).

Exploratory testing of group differences on prenatal paternal depression revealed no significant difference between the three groups (see Table 3).

#### Anxiety

Expectant fathers whose partners had inpatient treatment had the highest prevalence of moderate anxiety levels. Expectant fathers whose partner was prenatally distressed showed a distinct lower prevalence rate of moderate anxiety, as well as expectant fathers whose partner had no risk pregnancy (see Table 2). None scored above the cutoff for high levels of anxiety (cutoff ≥ 15).

**Fig. 2:**
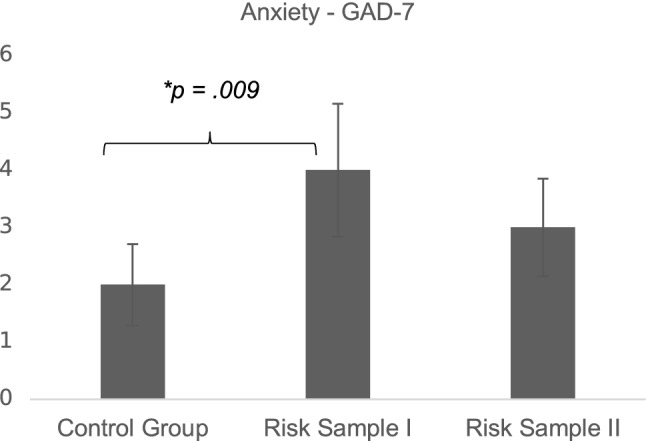
Group differences: median and confidence intervals of GAD-7

Exploratory testing of group differences showed a significant difference in anxiety level in the risk samples (see Table 2). Post hoc Mann–Whitney *U* tests were used to compare all pairs of groups, showing that expectant fathers whose partner had inpatient treatment are significantly more anxious than fathers whose partner had non-risk pregnancy (*U* = 1012, *z* =  − 2.61, *p* = 0.009; see Fig. 2).

#### Somatic symptom burden

For the pre-analysis, the influence of current paternal physical disease on paternal prenatal somatic symptom burden was tested using the Mann–Whitney *U* test. Results showed that expectant fathers with a current physical disease had no significant higher level of somatic symptom burden (Mdn = 8) compared to fathers without the current physical disease (Mdn = 5; *U* = 732, *p* = 0.489), indicating that current physical disease did not influence self-estimated somatic symptom burden. Thus, physical disease control is not necessary for the following statistical analyses.

Expectant fathers whose partner was prenatally distressed had the highest somatic symptom rates compared to the other two samples (see Table 2).

Exploratory testing of group differences on somatic symptom burden showed no significant difference between the three groups (see Table 3).

## Discussion

### Summary

This is the first study that examined and compared the prevalence rates of depression, anxiety, and somatic symptom burden of expectant fathers who face different risk situations during pregnancy. On a purely descriptive level, the data display a trend of higher symptom burden of depression, anxiety, and somatic symptoms in the two risk samples of expectant fathers indicating that men whose pregnant women were hospitalized (risk sample I) or suffered from prenatal depression (risk sample II) were more prenatally distressed. Exploratory testing of group differences indicated that expectant fathers whose partner was hospitalized due to prenatal medical complications were significantly more anxious than the control group of non-risk expectant fathers. The prevalence of clinically significant levels of anxiety was 12.2% in expectant fathers whose partner was hospitalized, which was almost three times higher compared to non-risk fathers (4.3%) and more than twice as high but not statistically significant compared to fathers with partners who suffered from prenatal depression (5.8%).

Even if not statistically significant, the depression prevalence rates showed similar patterns compared to anxiety prevalence. Consequently, the data indicate that expectant fathers with prenatally hospitalized partners do not only have a higher risk for anxiety disorders but also for depression compared to the control group and fathers with prenatally depressed partners. These results reflect the fact that expectant fathers are facing several additional burdening conditions while their pregnant partner had inpatient treatment due to severe pregnancy-related medical complications.

Somatization, instead, revealed the highest prevalence rate for medium and high levels of symptom burden in expectant fathers whose partners suffered from prenatal depression. The explanation for this inverted effect could be that men, whose pregnant partners are “only” psychologically distressed do not see themselves in the position to experience and express their strain on an open affective level which could imply emotional suppression, which is associated with more somatization [30]. Contrarily, men, who face the situation of their hospitalized pregnant partners, can refer to external stressors, which might make it easier for them to express their psychological strain on a more affective level [31].

### Former research

Without comparable studies focusing on paternal prenatal psychological and somatic symptom burden in specific risk samples, results are only seen about comparable studies of female perinatal risk samples. Our results indicate that fathers whose partner was hospitalized show the same risk for clinically significant anxiety (GAD-7 of 12.2%, cut-off > 9) as the hospitalized women themselves (GAD-7 of 13%, cutoff > 9) [32]; and a three time higher risk of developing anxiety disorder compared to the male norm population [33]. In addition, the rate of prenatal depression of fathers whose pregnant partner was hospitalized (19.5%) is comparable to the depression rate of hospitalized pregnant women which is between 12.5 and 44.2%, depending on the study methodology [34].

The prevalence of clinically significant depressive symptoms in non-risk fathers of 11.4% is comparable to the results of a recently published meta-analysis for prenatal paternal depression [35] well as to two recently published German studies, both using the EPDS with a cutoff value of 10 as a screening tool [6, 36]. Moreover, our examined prenatal depression rate of non-risk fathers is comparable to the prenatal depression rate of women without obstetric complications of a German sample (13.3%) [37]. This implies that expectant non-risk fathers have a comparable risk to develop depressive symptom burden as the pregnant women themselves.

The prevalence rates of medium levels of somatic symptom burden measured by the SSS-8 were considerably higher in the screened risk samples compared to the 10% prevalence rate in a German male population aged from 14 to 91 years [29]. The high somatic symptom rates detected in our study are interpreted as so-called “depressive equivalents” [38], indicating that men tend to express psychological burden in a more externalizing way and throughout somatization rather than on an affective level [39]. In addition, is the so-called “couvade syndrome,” which is understood as a male-specific psychosomatic phenomenon triggered by the partner’s pregnancy, mainly during the first and third trimester [7, 40]. Men who suffer from this syndrome develop non-specific physiological symptoms without a somatic cause but are highly correlated with depression and anxiety-related symptoms [7, 41].

### Limitations and directions for future research

As a limitation, selection bias has to be considered as there might be a pre-selection of participants who agreed to the study participation. It can be assumed that fathers-to-be who were interested in study participation might differ from those disagreeing in sense of openness and awareness of psychological issues in the perinatal period. Another limitation concerning the sample regards the recruitment of participants attending information evenings in the hospital. It can be assumed that especially expectant parents with high educational levels as well as a high willingness to be informed and perinatal engaged attend those evenings. Consequently, the results can only be interpreted with caution and should not be generalized. In addition, the comparatively low response rate in the risk samples could lead to selection bias. In addition, relatively small sample sizes were explored. Thus, former research should not only try to enhance the fathers’ response rate but also investigate bigger samples of expectant fathers facing prenatal risk situations to detect valid effects of prenatal risks on the fathers’ psychological well-being. Moreover, the design does not permit directional or causal conclusions to be drawn as there was only one time of measurement. Another limitation was that the level of prenatal female depressiveness in the non-risk sample was not controlled, which would allow a more profound result interpretation about risk sample I of women who suffer from prenatal depression. As this is the distinct differing factor between the non-risk sample and risk sample I, further research should include female prenatal depression of the non-risk pregnancy group as a control variable in the model. Further, it has to be noted that the EPDS is validated for men in the perinatal period [25, 26], but not in the German language as is the case for the use with women [42]. Thus, although we used the for men recommended cutoffs [25], the results have to be interpreted carefully.

Our study gained only explorative results on a purely descriptive level, thus future research is necessary to replicate our results on a confirmatory design.

## Conclusion and implications

Our findings indicate a significant concern in the prevalence of paternal prenatal psychological burden, especially in hospitalized pregnant partners due to obstetric complications. Next to depression and anxiety, our results imply that somatization is necessarily screened in the prenatal period as expectant fathers show up their psychological burden not only on a purely psychological level but also on a somatic one. Thus, the results underline the need for gynecologists to take concern not only about the mental health of the pregnant women themselves but also their counterparts. In line with former research, our data show that pregnancy is a critical life event that also affects fathers-to-be which gynecologists should be aware of and trained for. In Germany, the compulsory qualification “psychosomatic basic care” (Psychosomatische Grundversorgung) for future gynecologists should include the specificities of paternal mental health and should request them to also ask the men about their mental well-being e.g., while attending the women’s perinatal care. Giving male and female-specific screening questionnaires could simplify the first screening but should not replace face-to-face contact.

Further, specific prevention and treatment programs including fathers especially in risk situations like prenatal hospitalization should be developed and implemented in perinatal care. As psycho-social antenatal classes seem to be successful in reducing perinatal mental burden [44], psychoeducation on paternal and maternal perinatal mental health should be part of prenatal classes.
